# Are the [6]-coordinated sites in tourmaline in certain cases partially vacant?

**DOI:** 10.1007/s00710-023-00815-4

**Published:** 2023-02-22

**Authors:** Andreas Ertl

**Affiliations:** 1 Mineralogisch-Petrographische Abt., Naturhistorisches Museum, Burgring 7, A-1010 Wien, Austria; 2 Institut für Mineralogie und Kristallographie, Universität Wien, Josef-Holaubek-Platz 2, A-1090 Wien, Austria

**Keywords:** Tourmaline, Octahedral vacancies, Crystal structure, Crystal chemistry

## Abstract

Tourmaline has two different [6]-coordinated sites, the Y site and the Z site. Vacancies were reported from both sites. Based on high-quality chemical and single-crystal structural data it usually needs increasing proportions of short-range order configurations Na(Al_2_**□**)Al_6_(BO_3_)_3_[Si_6_O_18_]^V^(OH)_3_^W^(OH) or Na(Al_2_**□**)Al_6_(BO_3_)_3_[Si_6_O_18_]^V^(OH)_3_^W^F in order to produce Y-site vacancies (with **□** being the symbol for a vacant site). Less commonly, the short-range configuration Ca(Al_2_**□**)Al_6_(BO_3_)_3_[Si_5_T^3+^O_18_]^V^(OH)_3_^W^(OH) could occur in Al-rich tourmalines with a Si deficiency, where T^3+^  = B, Al. Therefore, tourmalines enriched in cations with charge 2 + (Fe^2+^, Mn^2+^, Mg) contain only insignificant Y-site vacancies. Aluminum-rich tourmalines with ≥ 7.0 apfu Al_total_ that usually contain ≥ 0.2 apfu Li may have significant vacancies at the Y site. However, no more than 12% vacancies (0.36 pfu) at the Y site can be observed in such samples. If no chemical data for Li is available it is proposed to calculate the Li content in such colourless or coloured tourmalines (elbaite, fluor-elbaite, fluor-liddicoatite, rossmanite) for Y = 2.8 apfu or for Y + Z + T = 14.8 apfu, because this calculation should give more accurate results than calculating the Li content as the difference to 3.0 apfu at the Y site. For Fe^2+^-rich and Mg-bearing tourmalines from the schorl-dravite series with MgO > 1.0 wt% (and only minor amounts of Fe^3+^, Cr^3+^ and V^3+^) the structural formula can still be calculated for Y + Z + T = 15 apfu, because such tourmalines do not appear to contain significant Y-site vacancies. It can further be concluded that the Z site could be only ≤ 1% vacant and therefore such vacancies would be insignificant even in Al-rich tourmaline.

## Introduction

The generalized formula of tourmaline-supergroup minerals can be written as XY_3_Z_6_(T_6_O_18_)(BO_3_)_3_V_3_W, as proposed by Henry et al. (2011). These authors and Hawthorne (1996, 2002) suggest occupancies by the following most common cations:

- ^IX^X = Na^+^, Ca^2+^, **□**
- ^VI^Y = Fe^2+^, Mg^2+^, Al^3+^, Li^+^, Mn^2+^, Fe^3+^, Cr^3+^, V^3+^
- ^VI^Z = Al^3+^, Mg^2+^, Fe^3+^, Cr^3+^, V^3+^
- ^IV^T = Si^4+^, Al^3+^, B^3+^
- ^III^B = B^3+^
- ^III^V = OH^–^, O^2–^
- ^III^W = OH^–^, F^–^, O^2–^

Some of these cations can be present simultaneously on two and even three structural sites, reflecting order–disorder phenomena, mainly between the octahedral Y- and Z-site occupants (Ertl et al. 2003 and references therein). The tourmaline supergroup currently comprises more than 40 valid mineral species accepted by the Commission on New Minerals, Nomenclature and Classification (CNMNC) of the International Mineralogical Association (IMA). They represent hydroxyl-, fluor- and oxy-species of X-site vacant, alkali, and calcic tourmalines with typical octahedral occupants as listed above (Henry et al. 2011). Crystal-chemical relations in the tourmaline supergroup and the crystal chemistry of tourmaline supergroup minerals have been investigated by many authors in the last 50 years (e.g., Donnay and Barton 1972; Povondra and Čech 1976; Deer et al. 1986; Hawthorne et al. 1993; Hawthorne 1996, 2002, 2016; Henry and Dutrow 1996; Ertl et al. 2002, 2012a, 2018; Bosi and Lucchesi 2004, 2007; Bosi et al. 2004, 2013, 2017; Hughes et al. 2004; Ertl and Tillmanns 2012; Ertl and Bačík 2020; Bačík and Fridrichová 2021). Tourmaline can also be a petrologic recorder of its geologic history as was shown by Van Hinsberg et al. (2011).

While vacancies at the [9]-coordinated X site are commonly well accepted because in some cases they may even dominate this site (foitite, oxy-foitite, magnesiofoitite, rossmanite, alumino-oxy-rossmanite), vacancies at the [6]-coordinated sites usually have generally not been characterized and described as well. Based on published high-quality data, the present work considers whether, why and when vacancies occur in the [6]-coordinated sites and in which tourmalines they would be expected. Any effects of such possible vacancies are also discussed.

## Previous work

The tourmaline crystal structure contains two different [6]-coordinated sites. Since more than 50 years, the site with the larger (distorted) octahedra is named the Y site and the site with the smaller (distorted) octahedra is named the Z site. Articles from the 1970ies and 1980ies, in which small amounts of Y-site vacancies, up to 0.16 per formula unit (pfu), have been cited, were summarized in Table 5 of Foit (1989). Many structural refinements in combination with chemical analyses indicate in later works that a minor Y-site vacancy could possibly exist (e.g., Hawthorne et al. 1993; Taylor et al. 1995; Ertl et al. 1997, 2003, 2006, 2009, 2010a, b).

Tourmaline samples of Mn-bearing fluor-elbaite, which were characterised chemically (including B_2_O_3_, Li_2_O, and H_2_O analysis) and by single crystal structure, show up to 0.21 pfu Y-site vacancies (Ertl et al. 2010a, b). Smaller Y-site vacancies up to ~ 0.10 pfu have been reported in in ^Y^Al-bearing schorl samples, which have also been fully characterised (Ertl et al. 2016). Higher Y-site vacancies (up to 0.19 pfu) have been reported from very Al-rich tourmalines with a dominant alumino-oxy-rossmanite component, i.e. with a vacancy-dominant X-site and with some tetrahedrally coordinated Al (Ertl et al. 2022). Even higher Y-site vacancies of 0.24 pfu were described from an Al-rich tourmaline, also with a vacancy-dominant X site, referred to as rossmanite (Ertl et al. 2009). Similar Y-site vacancies (up to 0.25 pfu) have been reported from B-rich olenite, also an Al-rich tourmaline (Ertl and Brandstätter 1998; Ertl et al. 2007, 2018). Interestingly, a Fe-rich and Mg-bearing tourmaline was described with 0.30 Y-site vacancies (Filip et al. 2012). These authors described a rather unusual tourmaline, which, in addition to Fe^2+^ and some Al at the Y-site, contains a relatively large amount of Fe^3+^. The samples with the highest reported Y-site vacancies are Al-rich tourmalines: a Mn- and Fe-bearing fluor-elbaite with 0.35 pfu vacancies (Grew et al. 2018) and a Fe-, Mn-, and Li-bearing olenite with 0.36 pfu vacancies (Ertl et al. 2012b). A natural Al-rich foititic tourmaline with ~ 0.35 pfu Y-site vacancies was also described (Wodara and Schreyer 2001). However, this sample lacks chemical analyses of B and H and no single-crystal structure refinement was performed.

In addition, from synthetic Al-rich tourmalines Y-site vacancies were described. Tourmalines, enriched in Al and Cu, showed up to 0.12 pfu Y-site vacancies (Ertl et al. 2015). Very Al- and B-rich tourmaline (olenite), a columnar crystal, was reported with ~ 0.17 pfu Y-site vacancies, which were determined by single-crystal structure refinement (Kutzschbach et al. 2016). Very Al- and B-rich olenite, synthesized by Wodara and Schreyer (1997), was described in an updated structural formula with 0.30 pfu Y-site vacancies (Ertl and Brandstätter 1998). The authors finally described X-site vacant and Al- and B-rich tourmaline with a structural formula containing ~ 0.29 pfu Y-site vacancies (Wodara and Schreyer 2001). Unfortunately, no single-crystal structure refinement was performed on the last two cited samples.

There are almost no publications describing Z-site vacancies. In an Al- and B-rich tourmaline (olenite), 0.08 pfu Z-site vacancies (for 6 Z sites) were reported, based on chemical and structural data (Hughes et al. 2000). These authors used a method of optimizing the site occupancies of cation sites in minerals with multiply occupied cation sites (Wright et al. 2000).

An extensive theoretical work by using bond-valence theory to explore the local arrangements involving vacancies at the Y and Z sites in the tourmaline structure was published by Bosi (2010). The results of this work show that arrangements involving tetrahedrally coordinated *R*^3+^-cations and octahedrally coordinated ^*Y*^*R*^2+^- and ^*Z*^*R*^2+^-cations around the oxygen atoms O8, O7 and O6 can be ruled out, together with arrangements involving vacancies and ^*Y*^Li^1+^. Based on bond-valence calculations Bosi (2010) concluded that vacancies are more favoured to occur at the Y site rather than at the Z site, in tandem with OH^−^ at the V and W sites, *R*^3+^ at the octahedral sites and Si^4+^ at the T site.

## Discussion

**Fig. 1:**
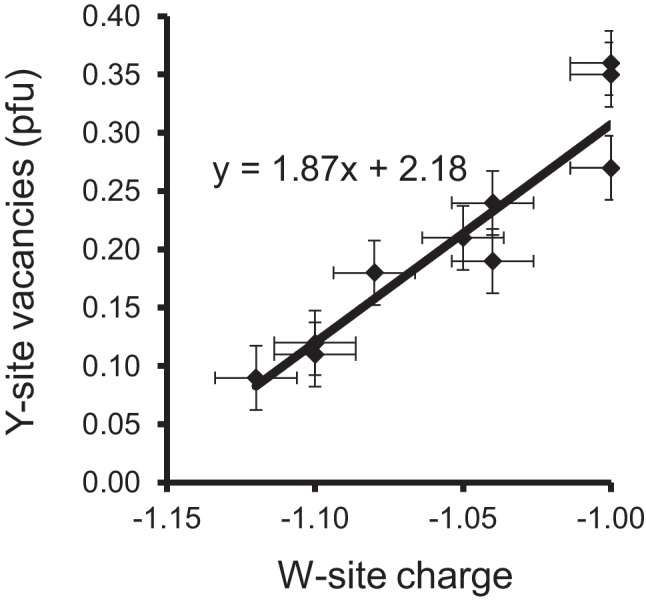
Correlation between the W-site charge and the Y-site vacancies for Al-rich tourmalines with ~ 6 apfu Si. All samples contain ≥ 5.6 apfu Si, 6.0 apfu at the Z site and MgO ≤ 0.02 apfu (Ertl et al. 2003, 2006, 2009, 2010a, 2012b; Grew et al. 2018)

**Fig. 2:**
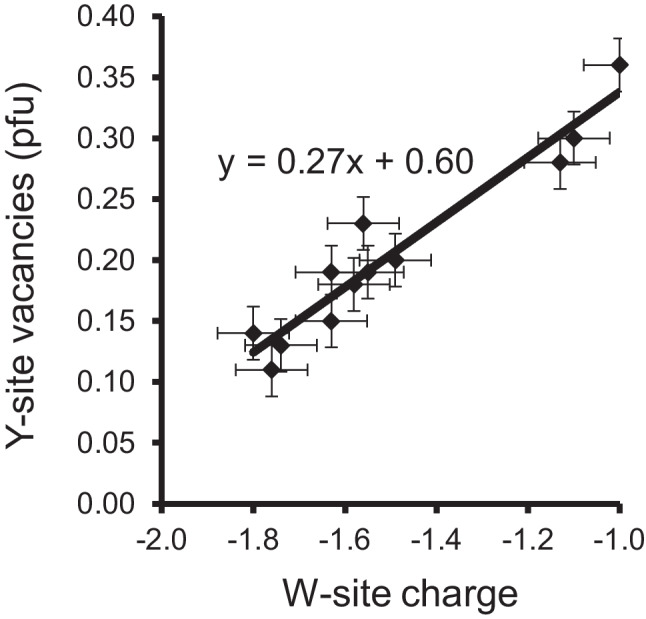
Correlation between the W-site charge and the Y-site vacancies for Al-rich tourmalines with ~ 5 apfu Si. All samples contain 4.5–5.5 apfu Si, 6.0 apfu Al at the Z site and MgO ≤ 0.2 apfu (Ertl and Brandstätter 1998; Schreyer et al. 2000; Kalt et al. 2001; Ertl et al. 2012a, 2022)

Usually, the best way to detect Y-site vacancies is when a tourmaline is fully characterised, including chemical analyses of the light elements B, Li, and H. If the tourmaline formula is normalized to 15 atoms per formula unit (apfu) for the sum of the occupants at the Y + Z + T sites, or for Y + Z + T + B = 18 apfu, it is clear that possible Y-site occupancies cannot be found. Tourmalines, which contain significant amounts of Mg, usually contain almost no Li and almost no tetrahedrally coordinated B. For such tourmalines, it is therefore not necessary to analyse B in most cases, since they usually contain exactly 3 apfu B. Since essentially Al-rich tourmaline samples with significant Y-site vacancies have been reported in the literature, only such samples were used for plotting correlations. Only the best-characterised tourmalines were used, most samples of which were analysed both chemically (including the light elements) and by single crystal X-ray diffraction. Some of the very Al-rich samples contain significantly less Si than 6 apfu. Hence, two different correlations were plotted, one with ~ 6 apfu Si (Fig. 1) and another with ~ 5 apfu Si (Fig. 2). Since the V site is filled with OH in most tourmalines and the W-site has a mixed occupancy of OH, F and O, it was of interest how the W-site charge interacts with the Y-site vacancies. If the W site has an occupancy of OH + F = 1, the W-site charge is -1. An oxy-component produces a lower W-site charge. If the W site were occupied only by oxygen, the W-site charge would be -2. Both figures show that the observed Y-site vacancy increases, when the oxy-component in tourmaline gets lower. This means that Al-rich tourmalines, where the W site is completely filled by OH + F, can have the highest Y-site vacancies. But when does the oxy-component increase in such samples? It increases when a tourmaline contains components of the endmembers darrellhenryite, dutrowite, lucchesiite, magnesio-lucchesiite, oxy-foitite, oxy-schorl or princivalleite, (not included here are V^3+^-, Cr^3+^-, and Fe^3+^-rich tourmalines). This means that the oxy-component at the W site usually increases, when such a tourmaline contains increasing amounts of (Fe^2+^, Mn^2+^, Mg^2+^, Li^1+^) and/or increasing X-site vacancies. Hence, the following substitutions can theoretically occur in Al-rich tourmalines to produce Y-site vacancies:

1. 2Y^2+^  + ^W^O =  > ^Y^Al^3+^  + ^Y^□ + ^W^(OH,F)
2. ^X^Ca^2+^  + 3Y^2+^  + ^W^O =  > ^X^Na^1+^  + 2^Y^Al^3+^  + ^Y^□ + ^W^(OH,F)
3. ^X^□ + Y^2+^  + ^W^O =  > ^X^Na^1+^  + ^Y^□ + ^W^(OH,F)
4. Y^2+^  + T^3+^  + ^W^O =  > ^Y^□ + ^T^Si^4+^ + ^W^(OH,F)
5. ^X^□ + ^Y^Al^3+^  + T^3+^  + ^W^O =  > ^X^Na^1+^  + ^Y^□ + ^T^Si^4+^  + ^W^(OH,F)
6. ^Y^Li + ^W^O =  > ^Y^□ + ^W^(OH,F)
7. ^X^Na^1+^  + 3Y^2+^  + ^T^Si^4+^  + ^W^O =  > ^X^Ca^2+^  + 2^Y^Al^3+^  + ^Y^□ + T^3+^  + ^W^(OH)
8. ^X^Ca^2+^  + 2^Y^Li^1+^  + ^Y^Al^3+^  + ^W^F =  > ^X^Na^1+^  + 2^Y^Al^3+^ + ^Y^□ + ^W^(OH)
  Y^2+^: Fe^2+^, Mn^2+^, Mg^2+^
  T^3+^: Al^3+^, B^3+^

**Fig. 3:**
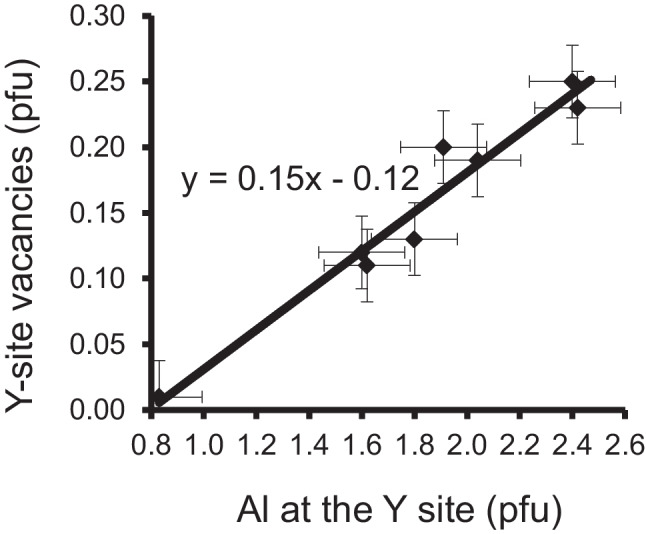
Correlation between Al content at the Y site and Y-site vacancies. All samples (oxy-schorl – olenite series with ^Z^Al_6.0_) are from Koralpe, Styria, Austria (Ertl and Brandstätter 1998; Kalt et al. 2001; Ertl et al. 2007)

Therefore, Mg-poor tourmalines with stoichiometric Si contents can have increasing Y-site vacancies when they are richer in Al and when they contain less (Fe^2+^, Mn^2+^ and Mg^2+^) cations, respectively. If such tourmalines exhibit low X-site vacancies, they will only have small amounts of ^[4]^Al and/or ^[4]^B. Such compositions will come close to a W-site occupancy of OH + F = 1. Regardless of whether tourmaline has a Si deficiency or not, it usually needs increasing proportions or short-range order configurations of Na(Al_2_**□**)Al_6_(BO_3_)_3_[Si_6_O_18_]^V^(OH)_3_ with OH or F at the W site in order to produce Y-site vacancies. A similar short-range order configuration (with OH at the W site) for Al- and Mn^2+^-rich tourmaline was proposed by Ertl et al. (2003). Later, for the elbaite-schorl series, short-range orders with (OH/F) at the W site were assumed (Ertl et al. 2010a). And indeed, a tourmaline was recently described that consists of 39% fluor-elbaite, 34% of the component Na^Y^(Al_2_□)^Z^Al_6_(BO_3_)_3_[Si_6_O_18_]^V^(OH)_3_^W^F, 16% fluor-tsilaisite and 11% fluor-schorl (Grew et al. 2018).

It is likely that these proposed substitutions depend not only on the chemistry of the whole rock, but also on the pressure and temperature conditions during the crystallization of tourmaline. Therefore, it would be helpful to have a larger number of tourmaline analyses of each locality. In general, complete published data of Al-rich tourmaline from a locality, including light elements and structural data, are not very common. Therefore, one is limited to check the proposed substitutions. There is, however, one locality from which such tourmalines have been extensively investigated. Therefore, the correlation between Al content at the Y site and Y-site vacancies was checked by using the Al-rich tourmalines of the Koralpe, Austria (Fig. 3). A strong positive correlation between Al content at the Y site and Y-site vacancies was observed. While the amount of Al^3+^ increases, the amount of Fe^2+^, Mg^2+^ and Si^4+^ decreases. Simultaneously, the amounts of cations with the charge 3 + (B, Al) increase at the tetrahedral site (Table 1 in Kalt et al. 2001). Therefore, it can be concluded that not only substitution (1) but also substitution (7) occurs. The latter substitution would require a short-range order configuration of Ca(Al_2_□)Al_6_(BO_3_)_3_[Si_5_T^3+^O_18_]^V^(OH)_3_^W^(OH), where T^3+^  = B, Al. This would be the only proposed substitution with a short-range order configuration, where the T site is not completely filled with Si^4+^. However, a calculation using bond-valence theory shows that this arrangement also appears to be stable.

**Fig. 4:**
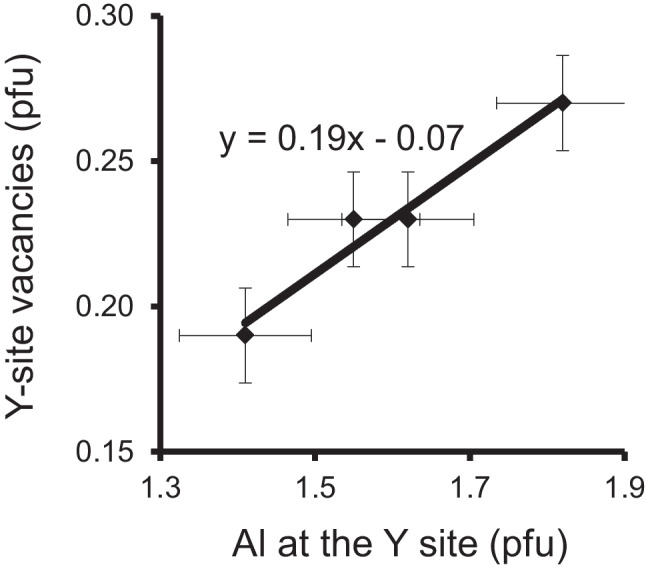
Correlation between Al content at the Y site and Y-site vacancies. All samples (fluor-liddicoatite – elbaite series with.^Z^Al_6.0_) are from Anjanabonoina, Madagascar (Ertl et al. 2006)

In tourmalines of the fluor-liddicoatite – elbaite series from Anjanabonoina, Madagascar, investigated by Ertl et al. (2006), the vacancies increase also with increasing Al content at the Y site as can be seen in Fig. 4. Because the amounts of Fe^2+^ and Mn^2+^ are extremely small in all tourmalines examined, it seems that the substitution (8) is mainly responsible for the Y-site vacancies. This is the only proposed substitution in which the W site is not occupied by oxygen but by fluorine. Interestingly, in substitution (8), an increasing Ca content does not lead to an increasing content of tetrahedrally coordinated B and Al, as in substitution (7). It can only be assumed that the tourmalines from Anjanabonoina, Madagascar crystallized at different temperatures and pressures than the tourmalines from the Koralpe, Austria. Anyway, such vacancies are unlikely to result from incorrect Li_2_O secondary ion mass spectrometry (SIMS) values, since the Li content is relatively low in many samples examined. The Al-richest sample from the study Ertl et al. (2006), which contains 0.89 apfu Li, yields 0.27 Y-site vacancies in the structural formula. The even more Al-enriched tourmalines (olenite samples) are actually lower in Li and therefore, assuming no Y-site vacancies, the calculated Li_2_O content would be much too high (> 30%) compared to the measured Li_2_O content for these samples. Furthermore, Al cannot fill these vacancies since Al has significantly more electrons than Li and therefore the calculated electron occupancy would be much too high compared to the observed electron occupancy at the Y site.

There are also other occupants than Al at the Y site that have a 3 + charge, such as Fe^3+^, Mn^3+^, Cr^3+^ and V^3+^. Apart from a sample with a relatively high content of Fe^3+^, described by Filip et al. (2012) it is not yet clear whether tourmalines enriched with these cations could also have Y-site vacancies. In order to find and understand further relationships in detail, however, more detailed investigations must be carried out.

## Conclusions

Based on high-quality available data, it can be concluded that significant Y-site vacancies can exist at least in Al-rich tourmalines. In particular, tourmalines with ≥ 7.0 apfu Al_total_ with usually ≥ 0.2 apfu Li may contain > 0.1 pfu vacancies at the Y site. However, no more than 12% vacancies (0.36 pfu) at the Y site appear to occur. It can be concluded that the calculation of the Li content by completely filling the Y site with Li when no chemical analysis is available is problematic for such Al-rich tourmalines from lithium pegmatites. The average Y-site vacancies in completely characterised Al-rich and Li-bearing (colourless – coloured) tourmalines (elbaite, fluor-elbaite, fluor-liddicoatite, rossmanite) is 0.185(7) (20 samples; Dyar et al. 1998; Ertl et al. 2006, 2009, 2010a, 2012b; Grew et al. 2018). Therefore, it is proposed to calculate the Li content in such tourmalines, if no chemical data is available, for Y = 2.8 apfu or Y + Z + T = 14.8 apfu or for Y + Z + T + B = 17.8 apfu. This calculation should give more accurate results than calculating the Li content as the difference to 3.0 apfu at the Y site.

Chemical data of Novák et al. (2004) showed that for (Fe,Mg)-rich, (Ca,Li,F)-poor tourmalines a W-site occupancy with [(OH,F)_0.5_O_0.5_] is more probable than with (OH,F)_1.0_, particularly in the tourmaline with a X-site vacancy > 0.3 pfu. Based on the trend seen in Fig. 1, no significant Y-site vacancies are expected in such tourmalines. Further investigations on Fe^2+^-rich and Mg-rich tourmalines have shown that samples with MgO > 1.0 wt% typically contain < 0.10 pfu Y-site vacancies (Bloodaxe et al. 1999; Ertl et al. 2012b). It cannot be ruled out that very small, calculated Y-site vacancies could be the result of random analytical errors propagating from measurements of oxides including light elements. Metamorphic tourmalines have > 0.2 apfu Mg and have very small amounts of Li, and thus, Li is typically an insignificant constituent in metamorphic tourmaline (Henry and Dutrow 1996). Therefore, no significant Y-site vacancies are expected in metamorphic tourmaline either. It can be concluded that for Fe^2+^-rich and Mg-bearing tourmalines with MgO > 1.0 wt% (and only minor amounts of Fe^3+^, Cr^3+^ and V^3+^) the structural formula can be calculated for Y + Z + T = 15 apfu, or for Y + Z + T + B = 18 apfu, because such tourmalines contain no significant Y-site vacancies.

It can be assumed that possible vacancies at the Z site could be ≤ 0.05 apfu (for 6 Z sites), which corresponds to ≤ 1% vacancies, and thus would be insignificant. The highest chance of detecting Z-site vacancies in tourmalines is in samples with a very large amount of [6]-coordinated cations with a 3 + charge, e.g., in Al-rich tourmalines. Further investigations will be necessary to obtain more high-quality data to be able to definitively prove Z-site vacancies. These conclusions are consistent with the theoretical approach of Bosi (2010) who, using bond-valence theory, predicted that vacancies occur at the Y site rather than the Z site.
